# Role of brassinosteroid signaling in modulating *Tobacco mosaic virus* resistance in *Nicotiana benthamiana*

**DOI:** 10.1038/srep20579

**Published:** 2016-02-03

**Authors:** Xing-Guang Deng, Tong Zhu, Xing-Ji Peng, De-Hui Xi, Hongqing Guo, Yanhai Yin, Da-Wei Zhang, Hong-Hui Lin

**Affiliations:** 1 Ministry of Education Key Laboratory for Bio-Resource and Eco-Environment, College of Life Science, State Key Laboratory of Hydraulics and Mountain River Engineering, Sichuan University, Chengdu, Sichuan, 610064, China; 2 Department of Genetics, Development and Cell Biology, Plant Science Institute, Iowa State University, Ames, IA 50011, USA

## Abstract

Plant steroid hormones, brassinosteroids (BRs), play essential roles in plant growth, development and stress responses. However, mechanisms by which BRs interfere with plant resistance to virus remain largely unclear. In this study, we used pharmacological and genetic approaches in combination with infection experiments to investigate the role of BRs in plant defense against *Tobacco Mosaic Virus* (TMV) in *Nicotiana benthamiana*. Exogenous applied BRs enhanced plant resistance to virus infection, while application of Bikinin (inhibitor of glycogen synthase kinase-3), which activated BR signaling, increased virus susceptibility. Silencing of *NbBRI1* and *NbBSK1* blocked BR-induced TMV resistance, and silencing of *NbBES1/BZR1* blocked Bikinin-reduced TMV resistance. Silencing of *NbMEK2, NbSIPK* and *NbRBOHB* all compromised BR-induced virus resistance and defense-associated genes expression. Furthermore, we found MEK2-SIPK cascade activated while BES1/BZR1 inhibited RBOHB-dependent ROS production, defense gene expression and virus resistance induced by BRs. Thus, our results revealed BR signaling had two opposite effects on viral defense response. On the one hand, BRs enhanced virus resistance through MEK2-SIPK cascade and RBOHB-dependent ROS burst. On the other hand, BES1/BZR1 inhibited RBOHB-dependent ROS production and acted as an important mediator of the trade-off between growth and immunity in BR signaling.

Plants and pathogens have engaged in an ongoing game of one-upmanship for millions of years. To survive from pathogen attack, plants have evolved a range of defense mechanisms to increase their tolerance. Phytohormones are increasingly recognized to play essential roles in plant-pathogen interactions. The stress related phytohormones salicylic acid (SA), jasmonic acid (JA), and ethylene (ET) are known to participate in defense responses to mitigate biotic stress in plants^1^,^2^. The signaling pathways of these hormones influence each other through a complex network of synergistic and antagonistic interactions^3^. In many cases, ET acts as a modulator of plant responses to either SA or JA. Newly emerging evidence suggest that some other plant hormones, such as abscissic acid (ABA), gibberellic acid (GA), cytokinins, auxins and brassinosteroids (BR), also play critical roles in plant-microbe interactions. These hormones render a positive or negative role in disease occurrence and interact with the SA-JA-ET signaling system^1^,^4^.

BRs are a class of steroid phytohormones that regulate many aspects of plant growth and development^5^. BR biosynthesis and signaling are well understood in *Arabidopsis*. In some crops, identification of a series of BR signaling components that are orthologous to those in *Arabidopsis*, suggesting that the BR signaling pathway is largely conserved among plants^6^. BRs are perceived by the plasma membrane-localized receptor BRASSINOSTEROID INSENSITIVE 1 (BRI1)^7^. Upon BR binding, BRI1 heterodimerizes with its co-receptor BRI1 ASSOCIATED KINASE 1 (BAK1)^8^, which leads to activation of BRI1 kinase activity. Activated BRI1 phosphorylates BR SIGNALING KINASE 1 (BSK1)^9^, which is followed by the phosphorylation and activation of BRI1 SUPPRESSOR 1 (BSU1). BSU1 inactivates a family of glycogen synthase kinase-3 (GSK3)^10^. This leads to dephosphorylation of BRI1 EMS SUPPRESSOR 1 (BES1) and BRASSINAZOLE RESISTANT 1 (BZR1), acting as major regulators of BR-induced transcriptional changes, which then become active^11^,^12^. Activation of BRI1 also results in phosphorylation and release of the receptor-like cytoplasmic kinase BOTRYTIS-INDUCED KINASE 1 (BIK1), which acts as a negative regulator of BR signaling^13^. In addition to its pivotal role in plant growth and development, BRs appear to protect plants from a variety of environmental stresses. There have been several reports describing the relationship between BRs and abiotic stress responses such as high or low temperature, drought, salinity and heavy metal contamination^14^,^15^,^16^,^17^. Several recent studies also reveal that BRs are involved in bacterial defense response^18^,^19^,^20^. However, it is unclear at the moment how BR signaling fit into virus resistance in plants.

Mitogen-activated protein kinase (MAPK) cascades are highly conserved signaling pathways that transduce extracellular stimuli into intracellular responses in eukaryotes. MAPK cascades are composed of three protein kinase modules: MAPK kinase kinases (MAPKKKs), MAPK kinases (MAPKKs) and MAPKs, which are linked in various ways to upstream receptors and downstream targets^21^. Plant MAPK cascades play pivotal roles in plant defense against pathogen attack. Two key MAPKs isolated from tobacco, wound-induced protein kinase (WIPK) and salicylic acid-induced protein kinase (SIPK) participate in *N*-gene-mediated resistance to *Tobacco mosaic virus* (TMV)^22^,^23^. Expression of *Nt*MEK2^DD^, a constitutively active form of a tobacco MAPKK upstream of WIPK and SIPK, induce hypersensitive response (HR)-like cell death in tobacco^24^,^25^. Similar to WIPK and SIPK, virus-induced gene silencing (VIGS) of several other MAPK components *NPK1* (MAPKKK), *MEK1* (MAPKK), or *NTF6* (MAPK) attenuate *N* gene- and *Pto*-mediated resistance against TMV^26^,^27^, indicating that the NPK1-MEK1-NTF6 pathway is another MAPK cascade involved in TMV resistance. These studies indicated that at least two MAPK cascades participated in disease resistance in tobacco plants.

In addition to the activation of MAPK cascades, another early biochemical event after plant sensing of invading pathogens is the generation of reactive oxygen species (ROS). Many studies reveal that ROS, especially H_2_O_2_ generated by NADPH oxidases encoded by *respiratory burst oxidase homolog* (*RBOH*) genes, play important roles in plant response to biotic and abiotic stresses^28^,^29^,^30^,^31^. In *Arabidopsis*, loss-of-function *RBOHD* and *RBOHE* mutants display decreased ROS production in response to infection with virulent *Pseudomonas syringae* pv. tomato DC3000^32^,^33^. Silencing *RBOHA* and *RBOHB* in *N. benthamiana* plants reduce ROS production and compromise resistance to *Phytophthora infestans*^28^. Meanwhile, ROS are also regulated by plant hormones such as ABA and BRs^17^,^34^. Recent studies report that elevation of ABA and BR levels result in increased production of hydrogen peroxide (H_2_O_2_) via RBOHs together with increased tolerance against a subset of abiotic stresses^35^,^36^.

In this study, we examined the roles of BR signaling pathway in modulating TMV resistance in *N. benthamiana*. Chemical treatment and VIGS approach demonstrated that BRI1, BSK1 and GSK3-like kinases positively while BES1/BZR1 negatively mediated BR-induced virus resistance. Loss-of-function analyses showed that MEK2-SIPK cascade and RBOHB played key roles in BR-induced virus resistance. We also showed that MEK2-SIPK cascade induced by BRs mediated RBOHB-dependent oxidative burst in *N. benthamiana* plants response to TMV.

## Results

### Foliar applications of BRs increase TMV resistance in *N. benthamiana* plants

**Figure 1:**
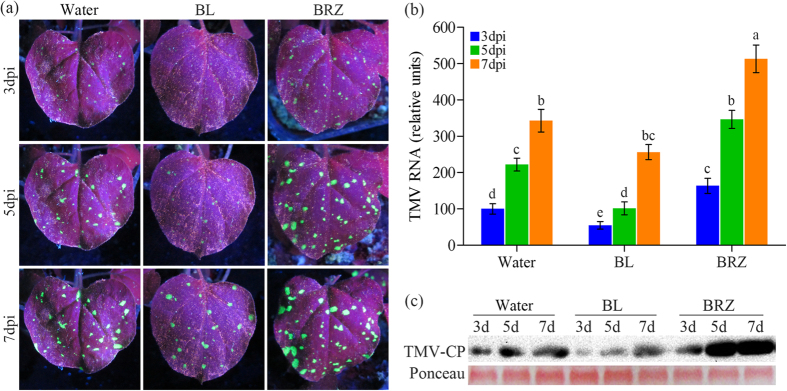
BRs increase TMV resistance in *N. benthamiana* plants. (**a**) Effects of foliar application of BL on plant defense against TMV-GFP infection. *N. benthamiana* leaves were treated with water, BL or BRZ and then inoculated with TMV-GFP. Pictures were taken under a UV light at 3, 5 and 7 days post inoculation (dpi). (**b**) Quantitative real-time PCR analysis of TMV mRNA accumulation levels in inoculated leaves collected at 3, 5 and 7 dpi, respectively. Bars represent mean and standard deviation of values obtained from three biological replicates per genotype and time point. Significant differences (P < 0.05) are denoted by different lowercase letters. (**c**) Western blotting analysis of coat protein accumulation of TMV in inoculated leaves collected at 3, 5 and 7 dpi. Rubisco proteins were used as loading controls and were stained by Ponceau.

We tested control and treated *N. benthamiana* plants for their resistance against infection of TMV, which was tagged with green fluorescent protein (GFP)^37^. *N. benthamiana* plants were pretreated with water, brassinolide (BL, the most active BR) and brassinazole (BRZ, a specific inhibitor of BR biosynthesis) before TMV-GFP inoculation. Virus accumulation was confirmed by direct observation of GFP fluorescence (Fig. 1a), as well as by quantitative real-time polymerase chain reaction (qRT-PCR) and western blotting analysis of viral replication (Fig. 1b,c) at 3, 5 and 7 days post-inoculation (dpi), respectively. *N. benthamiana* plants treated with BL showed weak GFP fluorescence as compared with water-treated plants (Fig. 1a). The conclusion is consistent with qRT-PCR and western blotting analysis of viral accumulation (Fig. 1b,c). However, plants treated with BRZ appeared to have the strongest GFP fluorescence (Fig. 1a) and the highest viral replication (Fig. 1b,c) level in comparison with water and BL treatment. These results indicate that BRs play a positive role in plant resistance to TMV.

### Effects of BR biosynthetic and signaling genes on BR-induced TMV defense

To further investigate at which level of the BR signaling pathway in limiting TMV infectivity, we used a *Tobacco rattle virus* (TRV) based VIGS system as a rapid genetics tool^37^,^38^ to silence BR biosynthetic and signaling genes in *N. benthamiana* plants and examined the functions in TMV infections. We targeted BR biosynthesis gene *NbDWARF* and several BR signaling components: *NbBRI1, NbBAK1, NbBSK1, NbBIK1, NbBSU1* and *NbBES1/BZR1*. These components were identified as the closest paralogs based on a BLAST search of the released genome sequence draft of *N. benthamiana* (http://solgenomics.net/organism/Nicotiana_benthamiana/genome) with *Arabidopsis* BR signaling components AtBRI1, AtBAK1, AtBSK1, AtBIK1, AtBSU1 and AtBES1/BZR1 (Fig. S1–S6). To investigate the involvement of these chosen genes in BR signaling in *N. benthamiana*, we evaluated BR sensitivity in silenced plants by measuring the growth phenotypes and the expression patterns of BR responsive genes after BL treatment. Control or BR signaling negative regulator *NbBIK1*-silenced plants were found to be responsive to 1 μM BL treatment, which showed excessive growth phenotypes, including increased leaf angles and petiole lengths. However, *NbBRI1*-, *NbBAK1*-, *NbBSK1*-, *NbBSU1*-, or *NbBES1/BZR1*-silenced plants did not respond to or had reduced responses to BL treatment (Fig. S7a,b). In *Arabidopsis*, BR signaling mediates feedback inhibition of the BR biosynthetic genes^10^. In control or *NbBIK1*-silenced *N. benthamiana* plants, expression of BR biosynthetic genes, including *NbCPD* and *NbDWARF*, were feedback-inhibited by BL treatment, while their expression were not decreased or decreased to a lesser extent in *NbBRI1*-, *NbBAK1*-, *NbBSK1*-, *NbBSU1*-, or *NbBES1/BZR1*-silenced plants (Fig. S7c,d). Taken together, the results shown here strongly indicate that these chosen genes are involved in the BRs response in *N. benthamiana*.

After 12 days of infiltration, the down-regulation of these chosen genes in silenced plants was confirmed by reverse transcription (RT)-PCR (Fig. S8). To confirm that silencing of these components was specific and did not affect transcript levels of related genes, we monitored the expression of their closest paralogs based on the genome sequence draft of *N. benthamiana*. Our results showed that these BR signaling components were specifically silenced without co-silencing their homologues (Fig. S8).

**Figure 2:**
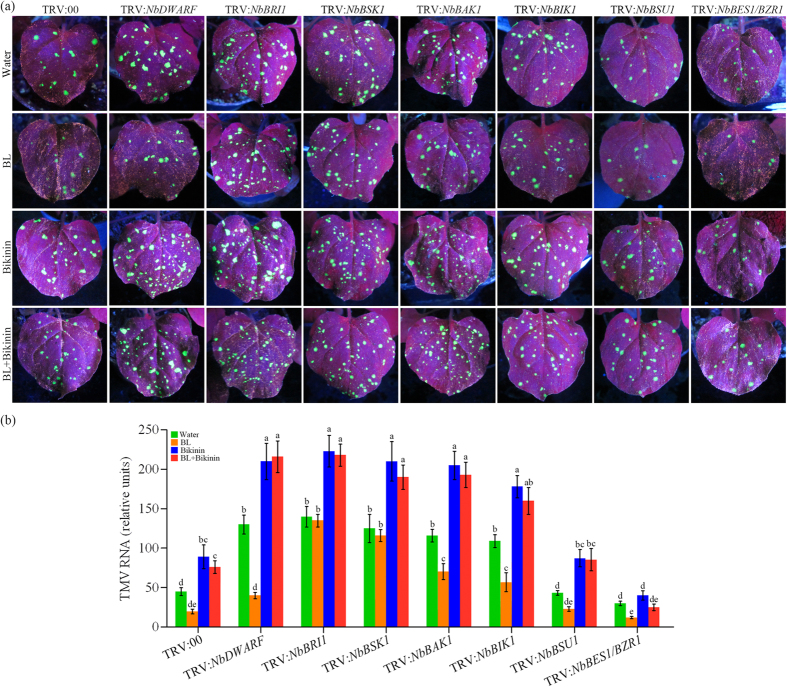
Effects of BR biosynthetic and signaling genes on BR-induced TMV defense. (**a**) Analysis of GFP fluorescence in the gene-silenced plants. The TMV-GFP spread assay was performed on *NbDWARF*-, *NbBRI1*-, *NbNSK1*-, *NbBAK1*-, *NbBIK1*-, *NbBSU1*- and *NbBES1/BZR1*-silenced and control *N. benthamiana* plants (TRV:00) pretreated with water, BL, Bikinin or BL + Bikinin. Photographs were taken from inoculated leaves at 5 days post inoculation (dpi). (**b**) Quantitative real-time PCR analysis of TMV mRNA accumulation levels in inoculated leaves collected at 5 dpi. Bars represent mean and standard deviation of values obtained from three biological replicates per genotype and time point. Significant differences (P < 0.05) are denoted by different lowercase letters.

Silenced plants were then inoculated with TMV-GFP and monitored for viral replication. As shown in Fig. 2, silencing of these genes, with the exception of *NbBSU1* and *NbBES1/BZR1*, resulted in decreased tolerance to TMV-GFP infection compared with the control (TRV:00) plants. Foliar application BL yielded a significant reduction in the GFP fluorescence and the levels of viral RNA in control, *NbDWARF*-, *NbBAK1-, NbBIK1-, NbBSU1*- and *NbBES1/BZR1*-silenced plants. However, silencing *NbBRI1* and *NbBSK1* substantially reduced the BR-induced TMV-GFP resistance compared with the control or other silenced plants. These results indicate that BRI1 and BSK1 are critical components for BR-induced virus resistance in *N. benthamiana*. Interestingly, treatment with the chemical Bikinin, which inhibit GSK3-like kinases^39^, resulted in increase of GFP fluorescence and the levels of viral RNA in TRV:00, TRV:*NbDWARF*, TRV:*NbBRI1*, TRV:*NbBSK1*, TRV:*NbBAK1* TRV:*NbBIK1* and TRV:*NbBSU1* plants, and BR-induced TMV-GFP resistance was largely inhibited by Bikinin treatment in these plants. However, the negative effects in virus resistance mediated by Bikinin were blocked in *NbBES1/BZR1*-silenced plants. Taken together, these results suggest that *NbBRI1* and *NbBSK1* are positive regulators in BR-induced virus resistance, and *NbBES1/BZR1* is a positive regulator in Bikinin-reduced virus resistance.

### BRs induce transcripts of RBOH and MAPK cascades after TMV infection

**Figure 3:**
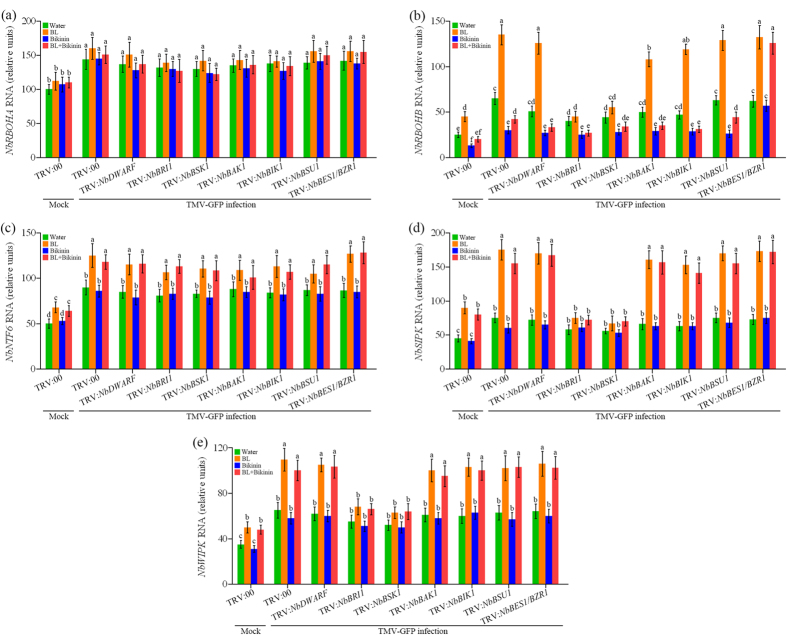
BRs induce transcripts of RBOH and MAPK cascades genes after TMV infection. Quantitative real-time PCR analysis expressions of *NbRBOHA* (**a**), *NbRBOHB* (**b**), *NbNTF6* (**c**), *NbSIPK* (**d**) and *NbWIPK* (**e**) in the gene-silenced or control plants pretreated with water, BL, Bikinin or BL + Bikinin at 2 days post inoculation (dpi) with TMV-GFP infection. “Mock” means seedlings not infected with TMV-GFP. Bars represent mean and standard deviation of values obtained from three biological replicates per genotype and time point. Significant differences (P < 0.05) are denoted by different lowercase letters.

Previously studies showed that both RBOH and MAPK pathways were involved BR-induced abiotic stress tolerance^35^. To investigate whether these pathways are required for BR-induced TMV resistance in *N. benthamiana*. We tested the expression of *NbRBOHA, NbRBOHB* (RBOH pathway) and *NbNTF6, NbSIPK, NbWIPK* (MAPK cascades pathway) using qRT-PCR. As shown in Fig. 3, all these genes were significantly activated in *N. benthamiana* plants at 2 dpi with TMV-GFP inoculation compared with mock-inoculated plants, indicating their involvement in plants response to TMV. The expression of *NbRBOHB* (Fig. 3b), *NbNTF6* (Fig. 3c), *NbSIPK* (Fig. 3d) and *NbWIPK* (Fig. 3e) were up-regulated more significantly after BL treatment. However, the expression of *NbRBOHA* (Fig. 3a) was not significantly altered after BL treatment in comparison with water-treated plants. From these data, we can conclude that BRs induce transcripts of *NbRBOHB, NbNTF6, NbSIPK* and *NbWIPK* in *N. benthamiana*. Furthermore, transcripts of *NbRBOHB, NbSIPK* and *NbWIPK* increased slightly in *NbBRI1* or *NbBSK1*-silenced plants after foliar applications of BL, indicating that BRs regulate these genes expression through BRI1 and BSK1. Interestingly, Bikinin treatment reduced transcript levels of *NbRBOHB* and BR-induced *NbRBOHB* expression in TRV:00, TRV:*NbDWARF*, TRV:*NbBRI1*, TRV:*NbBSK1*, TRV:*NbBAK1* TRV:*NbBIK1* and TRV:*NbBSU1* plants. Again, these suppression effects were compromised in *NbBES1/BZR1*-silenced plants.

### Involvement of ROS in BR-induced TMV defense

**Figure 4:**
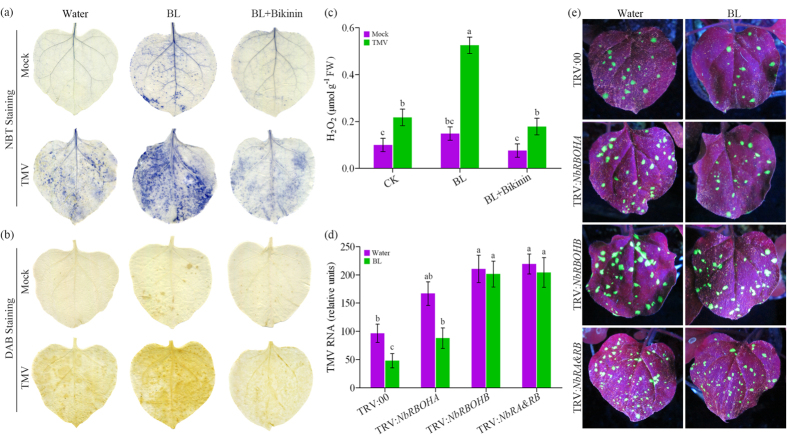
Involvement of ROS in BR-induced TMV defense. NBT- (**a**) and DAB (**b**)-stained mock or TMV-GFP inoculated *N. benthamiana* leaves pretreated with water, BL or Bikinin at 2 days post inoculation (dpi). (**c**) H_2_O_2_ levels in mock or TMV-GFP inoculated leaves determined at 2 dpi. (**d**) Quantitative real-time PCR analysis of TMV mRNA accumulation levels in *NbRBOHA*-, *NbRBOHB*-, *NbRBOHA*&*RBOHB*-silenced and control plants (TRV:00) pretreated with water or BL at 5 dpi. (**e**) TMV-GFP spread in gene-silenced leaves pretreated with water or BL was photographed at 5 dpi. “Mock” means seedlings not infected with TMV-GFP. Bars represent mean and standard deviation of values obtained from three biological replicates per genotype and time point. Significant differences (P < 0.05) are denoted by different lowercase letters.

ROS act as second messengers in stress response^17^. To determine a possible role of ROS in BR-induced virus resistance in *N. benthamiana*, we attempted to detect *in situ* accumulation of superoxide (O_2_^−^) and hydrogen peroxide (H_2_O_2_) using nitroblue tetrazolium (NBT) and 3,3′-diaminobenzidine (DAB) staining procedures, respectively. Both procedures detected increased staining in BL-treated leaves relative to that in water-treated leaves, although both of them increased under TMV-GFP infection conditions (Fig. 4a,b). We further determined H_2_O_2_ levels in these leaves. Similarly, in BL-treated leaves, H_2_O_2_ content was significantly higher than those of in the water-treated leaves infected with TMV-GFP (Fig. 4c). These results reveal that BRs can induce ROS generation in response to TMV infection. Importantly, BR-increased ROS accumulation was largely inhibited again by Bikinin treatment (Fig. 4a–c).

NADPH oxidase is an important source of apoplastic H_2_O_2_ accumulation^40^. To determine whether BR-induced virus defense is related to NADPH oxidase (*RBOH*) genes, we compared TMV resistance in *NbRBOHA*-, *NbRBOHB*- and *NbRBOHA*&*RBOHB*-silenced plants. All these silenced plants showed more susceptible to TMV-GFP infection, as indicated by the increased levels of viral RNA (Fig. 4d) and the increased green fluorescence (Fig. 4e) compared with the TRV:00 inoculated plants. Furthermore, BL pre-treatment clearly increased TMV resistance in control and *NbRBOHA*-silenced plants, but it had little effect in *NbRBOHB*- and *NbRBOHA*&*RBOHB*-silenced plants (Fig. 4c,d). These results suggest that RBOHB-dependent oxidative burst plays an essential role in the BR-induced TMV resistance in *N. benthamiana*.

### Effects of MAPK cascades in BR-induced TMV defense

There are two MAPK cascades in *N. benthamiana* plants, MEK1-NTF6 and MEK2-WIPK/SIPK pathways^41^. To determine whether these pathways are involved in BR-induced virus resistance, we knocked down the genes *NbMEK1, NbNTF6, NbMEK2, NbWIPK* and *NbSIPK* using VIGS. The silencing effects on TRV:*NbMEK1*, TRV:*NbNTF6*, TRV:*NbMEK2*, TRV:*NbWIPK* and TRV:*NbSIPK* plants were confirmed by comparing their expression levels with TRV:00 control plants (Fig. S8).

**Figure 5:**
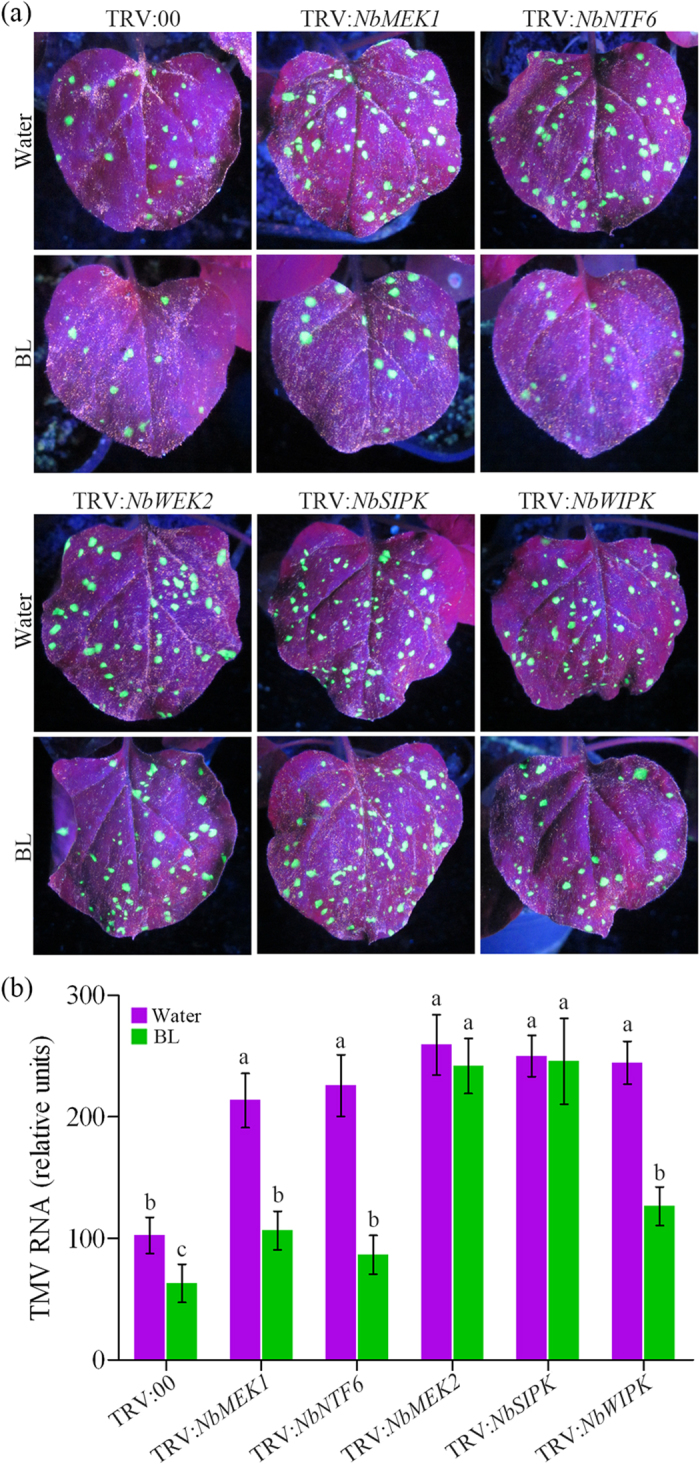
Effects of MAPK cascades in BR-induced TMV defense. (**a**) TMV-GFP spread was performed in *NbMEK1*-, *NbNTF6*-, *NbMEK2*-, *NbSIPK*-, *NbWIPK*-silenced and control *N. benthamiana* plants (TRV:00) pretreated with water or BL. Photographs were taken from inoculated leaves at 5 days post inoculation (dpi). (**b**) Quantitative real-time PCR analysis of TMV mRNA accumulation levels in inoculated leaves collected at 5 dpi. Bars represent mean and standard deviation of values obtained from three biological replicates per genotype and time point. Significant differences (P < 0.05) are denoted by different lowercase letters.

These silenced *N. benthamiana* plants were then inoculated with TMV-GFP and monitored for viral replication. Extensive green fluorescence was observed in all silenced leaves than in control leaves, which displayed a few fluorescent area (Fig. 5a). Similar results were observed when a qRT-PCR was performed to detect viral mRNA levels (Fig. 5b). Furthermore, BL treatment could reduce TMV-GFP accumulation in control, TRV: *NbMEK1*, TRV:*NbNTF6* and TRV:*NbWIPK* plants, but not in TRV:*NbMEK2* and TRV:*NbSIPK* plants (Fig. 5a,b). These results suggest that both MEK1-NTF6 and MEK2-WIPK/SIPK pathways were involved virus immunity in *N. benthamiana*, and only MEK2-SIPK cascade is necessary for BR-induced virus resistance.

### Inhibition of MEK2-SIPK cascade compromises BR-induced RBOHB-dependent oxidative burst after TMV infection

**Figure 6:**
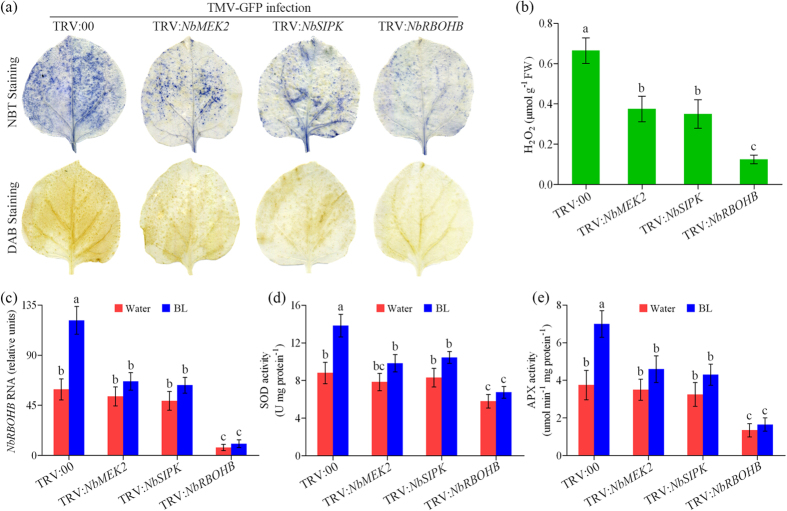
Silencing *NbSIPK* compromises BR-induced RBOHB-dependent oxidative burst after TMV infection. (**a**) NBT- and DAB-stained TMV-GFP infected leaves collected from *NbMEK2*-, *NbSIPK*-, *NbRBOHB*-silenced and control *N. benthamiana* plants (TRV:00) pretreated with BL. (**b**) H_2_O_2_ levels in TMV-GFP inoculated leaves pretreated with BL. (**c**) Quantitative real-time PCR analysis of *RBOHB* mRNA levels in gene-silenced plants pretreated with water or BL. The activities of the antioxidant enzymes SOD (**d**) and APX (**e**) in gene-silenced plants pretreated with water or BL at 2 days post inoculation (dpi) with TMV-GFP inoculation. Bars represent mean and standard deviation of values obtained from three biological replicates per genotype and time point. Significant differences (P < 0.05) are denoted by different lowercase letters.

We have shown that both RBOHB and SIPK were required for BR-induced TMV resistance. Therefore, the effect of MEK2-SIPK cascade on BR-induced RBOHB-dependent oxidative burst after TMV-GFP infection was investigated in *N. benthamiana*. We first examined ROS accumulation and transcript of *NbRBOHB* gene in TRV:00, TRV:*NbMEK2*, TRV:*NbSIPK* and TRV:*NbRBOHB* plants (pretreated with BL) at 2 dpi with TMV-GFP inoculation. A decrease of O_2_^−^ and H_2_O_2_ accumulation was observed not only in TRV:*NbRBOHB* plants, but also in TRV:*NbMEK2* and TRV:*NbSIPK* plants, when compared with that in TRV:00 control plants (Fig. 6a,b). The reduction of ROS accumulation in TRV:*NbRBOHB* plants was more obviously as compared to TRV:*NbMEK2* and TRV:*NbSIPK* plants. Similar to the ROS content, BL treatment up-regulated *NbRBOHB* transcript significantly in TRV:00 plants but not in TRV:*NbRBOHB*, TRV:*NbMEK2* and TRV:*NbSIPK* plants (Fig. 6c). We then determined the activities of Superoxide dismutase (SOD) and Ascorbate peroxidase (APX) in these plants. BL treatment also caused significant increases in the total activities of SOD and APX in control plants but not in TRV:*NbRBOHB*, TRV:*NbMEK2* and TRV:*NbSIPK* plants at 2dpi with TMV-GFP inoculation (Fig. 6d,e). Taken together, these data demonstrate that MEK2-SIPK cascade is required for BR-induced RBOHB-dependent oxidative burst in *N. benthamiana* plants response to TMV.

### Overexpression of *SIPK* enhances BR-induced oxidative burst and virus resistance

To further determine the role of SIPK in BR-induced oxidative burst in response to TMV infection, gain-of-function analyses of SIPK was done using *Agrobacterium* infiltration methods. Leaves of TRV:00, TRV:*NbRBOHB* and TRV:*NbSIPK N. benthamiana* plants (pretreated with BL) were infiltrated with *Agrobacterium* carrying 35S:*SIPK*-*Flag* construct. We also included the empty vector (35S:00) as a negative control. The expression of SIPK was confirmed by immune-blot analysis using anti-*Flag* antibody (Fig. S9).

**Figure 7:**
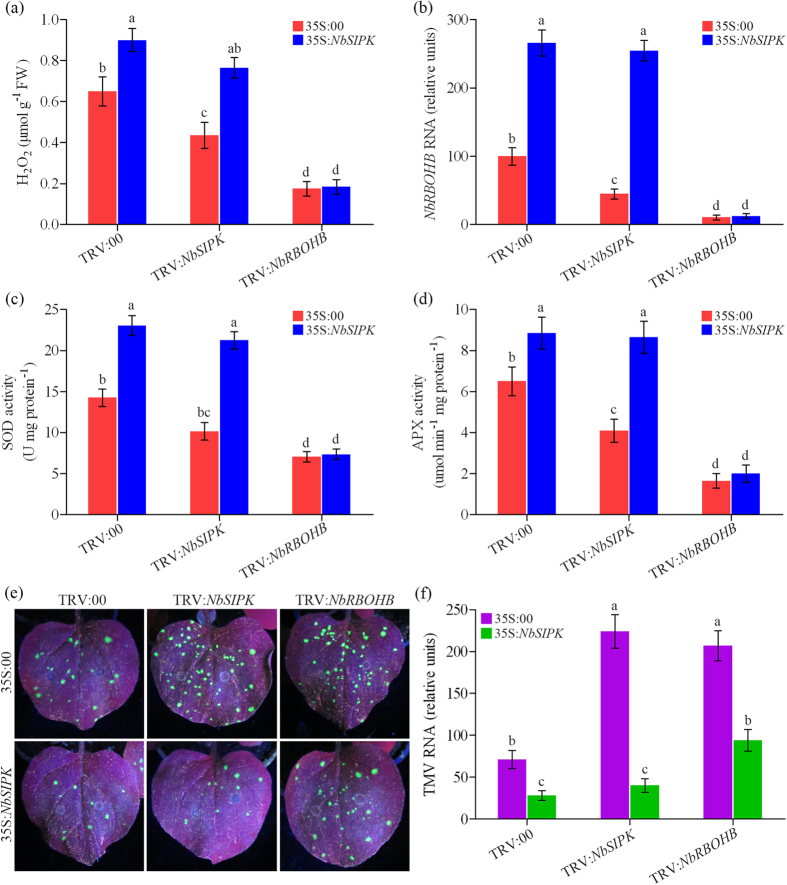
Transient expression of SIPK enhances BR-induced oxidative burst and virus resistance. H_2_O_2_ levels (**a**), expression of *NbRBOHB* (**b**) and activities of the antioxidant enzymes SOD (**c**) and APX (**d**) in *NbSIPK*-, *NbRBOHB*-silenced and control (TRV:00) *N. benthamiana* plants (pretreated with BL and inoculated with TMV-GFP) which were infiltrated with 35S: *NbSIPK* or 35S:00. (**e**) TMV-GFP spread was performed in treated plants as described in (**a–d**). Photographs were taken from inoculated leaves at 5 days post inoculation (dpi). (**f**) Quantitative real-time PCR analysis of TMV mRNA accumulation levels in inoculated leaves collected at 5 dpi. Bars represent mean and standard deviation of values obtained from three biological replicates per genotype and time point. Significant differences (P < 0.05) are denoted by different lowercase letters.

Our results showed that transient expression of *NbSIPK* substantially increased H_2_O_2_ accumulation in TRV:00 and TRV:*NbSIPK N. benthamiana* plants infected with TMV-GFP, but not in TRV:*NbRBOHB* plants as compared with control plants (Fig. 7a). Similar to H_2_O_2_ contents, transcript level of *NbRBOHB*, activities of SOD and APX were induced significantly by the expression of SIPK in TRV:00 and TRV:*NbSIPK* plants, while these increasing effects were compromised in TRV:*NbRBOHB* plants (Fig. 7b–d). In addition, transient expression of SIPK also yielded a significant reduction in the GFP fluorescence (Fig. 7e) and levels of viral RNA (Fig. 7f) in TRV:00 and TRV:*NbSIPK* plants but less obviously in TRV:*NbRBOHB* plants. Taken together, these results confirm the role of SIPK in BR-induced oxidative burst and virus resistance, and SIPK probably acts upstream of RBOHB.

### BRs activate defense-associated genes expression after TMV infection

**Figure 8:**
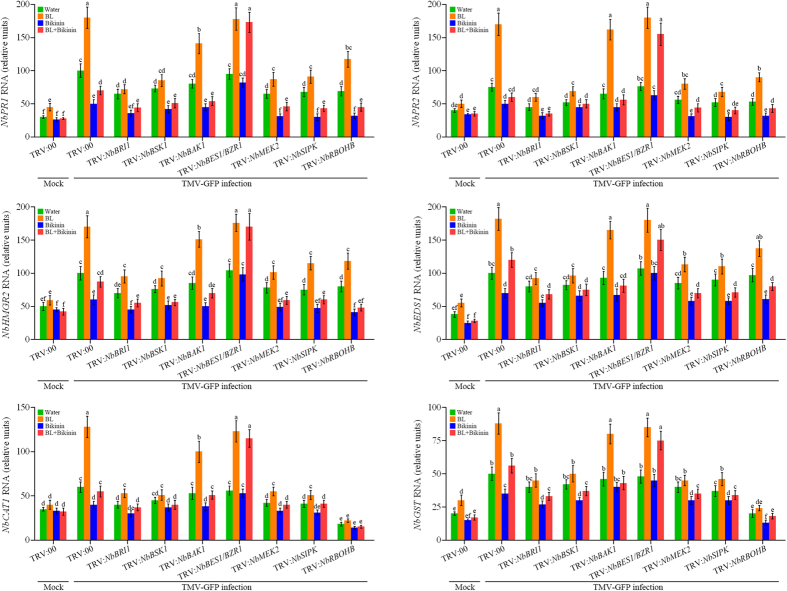
Relative expression of disease-related genes (*PR1, PR2, HMGR2* and *EDS1*) and antioxidant-related genes (*CAT1* and *GST*) in *NbBRI1*-, *NbBSK1, NbBAK1, NbBES1/BZR1*-, *NbMEK2*-, *NbSIPK*-, *NbRBOHB*-silenced and control (TRV:00) *N. benthamiana* plants at 2 days post inoculation (dpi) with TMV-GFP infection, these plants were pretreated with water, BL, Bikinin or BL + Bikinin. “Mock” means seedlings not infected with TMV-GFP. Bars represent mean and standard deviation of values obtained from three biological replicates per genotype and time point. Significant differences (P < 0.05) are denoted by different lowercase letters.

To further analyze the underlying molecular mechanisms of BR-induced virus resistance, we examined the effects of BR levels on expression of several genes involved in the defense response. Transcripts of four disease-related genes (*NbPR1, NbPR2, NbHMGR2* and *NbEDS1*) and two antioxidant-related genes (*NbCAT1* and *NbGST*) were detected. As shown in Fig. 8, transcripts of all these genes were significantly induced by BL treatment in TRV:00 plants (as the control). Importantly, silencing of *NbBRI1, NbBSK1, NbMEK2, NbSIPK* and *NbRBOHB* largely compromised BL-induced up-regulation of these defense genes, but in *NbBES1/BZR1*-silenced plants BL still up-regulated transcripts of these genes. It is worth noting that silencing of *NbBAK1* inhibited BL-induced up-regulation of *NbPR1* and *NbCAT1* to a lesser extent, confirming that this component played a relative smaller role in BR-induced immunity signaling (Fig. 8). Furthermore, Bikinin treatment decreased the transcripts of these six genes in all plants, while in *NbBES1/BZR1*-silenced plants, Bikinin failed to down-regulate transcripts of these genes. However, silencing of *NbDWARF, NbBSU1, NbBIK1, NbRBOHA, NbMEK1, NbNTF6* and *NbWIPK* had little effect on BL-mediated up-regulation of all the six genes in *N. benthamiana* plants (Fig. S10). In addition, Bikinin treatment inhabited BR-induced up-regulation of the six genes, and the inhibition effects were compromised in *NbBES1/BZR1*-silenced plants.

## Discussion

Recent studies indicate that besides their critical role in orchestrating growth and developmental processes, BRs are also implicated in plant responses to pathogen attack^6^,^42^. We previously reported that BRs could induce resistance against *Cucumber mosaic virus* in *Arabidopsis*^43^. However, the role of BRs in plant defense and the mechanisms of their actions are not well understood, and even controversial. The research described here aims to provide a further characterization of the role of BR-mediated defense signaling using a *N. benthamiana* and TMV-GFP interaction system. Through the well-established TRV-based VIGS approach, we reveal that the BR signaling pathway, MAPK cascades, and NADPH oxidase play important roles in BR-mediated TMV defense in *N. benthamiana*.

In recent years, rapid progress has been made in elucidating the BR signaling pathway in *Arabidopsis*^10^. In *N. benthamiana*, however, only one counterpart of the *Arabidopsis* BR signaling component has been identified (NbBAK1)^18^. No additional BR signaling components have been characterized, and little is known about the downstream events of BR signal transduction in *N. benthamiana*. Here a series of BR signaling components were identified based on *Arabidopsis* homologues in *N. benthamiana*. Protein sequences alignment, BR-regulated growth phenotypes and gene expression studies confirmed that these components play important roles in BR responses in *N. benthamiana*, similar to *Arabidopsis* (Fig. S1–S7). Our study further showed that silencing BR biosynthetic and signaling genes *NbDWARF, NbBRI1, NbBSK1, NbBAK1* and *NbBIK1* increased susceptible to TMV-GFP infection in *N. benthamiana* plants (Fig. 2), suggesting these components participated in anti-viral immunity. To date, most studies aimed at understanding how BRs mold pathological outcomes have focused on the role of BAK1. Besides its role in BR signaling, BAK1 is also involved in the regulation of microbe-induced cell death, and interact with various pattern recognition receptors (PRRs), including the flagellin receptor FLS2, to drive pathogen-triggered immunity (PTI)^18^. Several studies showed that BAK1’s function in innate immunity is independent of its function in BR signaling and BRs can act on plant defenses independently of BAK1^19^,^20^. Recently, BIK1 is also added to the list of signaling components shared by the BR and PTI pathways, although BIK1 negatively regulates the BR-signaling pathway and positively regulates the FLS2–PTI signaling^13^, its functions in both processes are mechanistically uncoupled. BSK1 has been reported to function as a positive regulator of flg22-induced ROS production and SA accumulation by physically interacting with FLS2, and inhibition of BSK1 increase susceptibility to both virulent and avirulent pathogens in *Arabidopsis*^44^. In this study, we showed that BR-induced virus resistance and defense-associated genes expression were largely compromised in *NbBRI1* and *NbBSK1*-silenced plants (Figs 2 and 8). These results suggest that an intact BR receptor complex/early cascade is required in BR-mediated virus resistance signaling.

ROS, especially H_2_O_2_ play an indispensable role in signal recognition and transduction in plant responses to biotic and abiotic stresses^31^,^40^. Recent studies indicate that BR-induced ROS accumulation enhances plant tolerance to abiotic stress^16^,^17^,^35^,^36^. However, there is no report about a connection between ROS and BR-induced virus defense so far. In the present study, we revealed the function of ROS in BR-induced virus defense. Exogenously applied BL up-regulated the accumulation of ROS in *N. benthamiana* leaves infected with TMV-GFP (Fig. 4a–c), suggesting that ROS was very likely to participate in BR-induced virus defense signaling. NADPH oxidase is a main source of H_2_O_2_ accumulation^31^. Here, we also found that BL treatment induced the expression of NADPH oxidase gene *NbRBOHB* in *N. benthamiana* (Fig. 3a,b). Again, BL treatment failed to increase the tolerance to TMV in *NbRBOHB*-silenced plants, but still effective in enhancing the tolerance in *NbRBOHA*-silenced plants (Fig. 4e). These results suggest that BR-induced RBOHB-dependent H_2_O_2_ production is not only involved in plant tolerance to abiotic stresses, but also involved in resistance to virus.

MAPK cascades are known as major pathways by which extracellular stimuli are transduced into intracellular responses in plants. The requirement of these kinases in defense-related signaling has been demonstrated previously in the *Pto, N* gene-mediated, gene-for-gene interaction and PTI pathways^23^,^43^,^45^. A subset of MAPKs in plants, represented by tobacco SIPK/WIPK and *Arabidopsis* MPK3/MPK6, are implicated in regulation of defense hormone (SA, JA and ET) biosynthesis and the signaling processes^46^,^47^,^48^. Recent studies also demonstrate a link between BRs and MAPK cascades. MKK4 and MKK5 act downstream of BR signaling as targets of the BIN2 kinase in *Arabidopsis*^49^. BRs regulate stomatal development by activating the MAPK cascade^50^. Inhibiting the expression and activity of MAPKs compromises BR-induced stress tolerance. Here, we identified a link between MAPK cascades and BR-mediated virus defense response. Our results showed that silencing of *NbMEK1, NbNTF6, NbMEK2, NbWIPK* and *NbSIPK* in *N. benthamiana* plants reduced tolerance to TMV-GFP (Fig. 5), suggesting that both MEK1-NTF6 and MEK2-WIPK/SIPK cascades were involved in plant resistance against virus. Although BRs increased the transcripts of *NbNTF6, NbWIPK* and *NbSIPK* in different degrees (Fig. 3c–e), the hormones still enhanced the tolerance against TMV infection in *NbMEK1*-, *NbNTF6*-, and *NbWIPK*-silenced plants, but not in *NbMEK2*- and *NbSIPK*-silenced plants. All these results suggest that in *N. benthamiana* the MEK2-SIPK cascade is required in BR-induced virus resistance.

Previous studies have revealed that there is an interesting relationship between NADPH oxidase-produced ROS and MAPK activation in plants exposed to various stresses^35^. Pathogen-responsive MAPKs are believed to function downstream of early ROS burst in plant immunity signaling, because defense-related MAPKs, including *Arabidopsis* MPK3, MPK6 and MPK4, or tobacco SIPK and WIPK, can be activated by exogenously application of H_2_O_2_^51^. There is also evidence suggesting that acclimation-induced H_2_O_2_ production can activate MAPKs in tomato^35^. However, recent evidence suggest that MAPK activation is independent of the NADPH oxidase-mediated oxidative burst, and MAPKs may act upstream of ROS burst. In *N. benthamiana*, silencing of *NbSIPK* and *NbNTF6* can suppress INF1 elicitin-induced RBOHB expression and ROS accumulation^52^, and overexpression of *NbSIPK* enhances sensitivity to stress-induced ROS^53^. In *Arabidopsis*, conditional activation of MPK3 and MPK6 induces ROS-dependent callose deposition, whereas inactivation of MPK3/MPK6 diminishes ROS accumulation^54^. In the present study, silencing of *NbMEK2* and *NbSIPK* arrested while transient expression of *NbSIPK* enhanced the RBOHB-dependent oxidative burst induced by BRs (Figs 6 and 7). These results suggest that in BR-induced virus defense signaling, MEK2-SIPK cascade regulate the early oxidative burst resulting from the induction of *NbRBOHB* expression. We also found BRI1 and BSK1 functioned upstream of SIPK and RBOHB because silencing of *NbBRI1* or *NbBSK1* compromised BR-induced the expression of *NbSIPK* and *NbRBOHB* (Fig. 3b,d).

**Figure 9:**
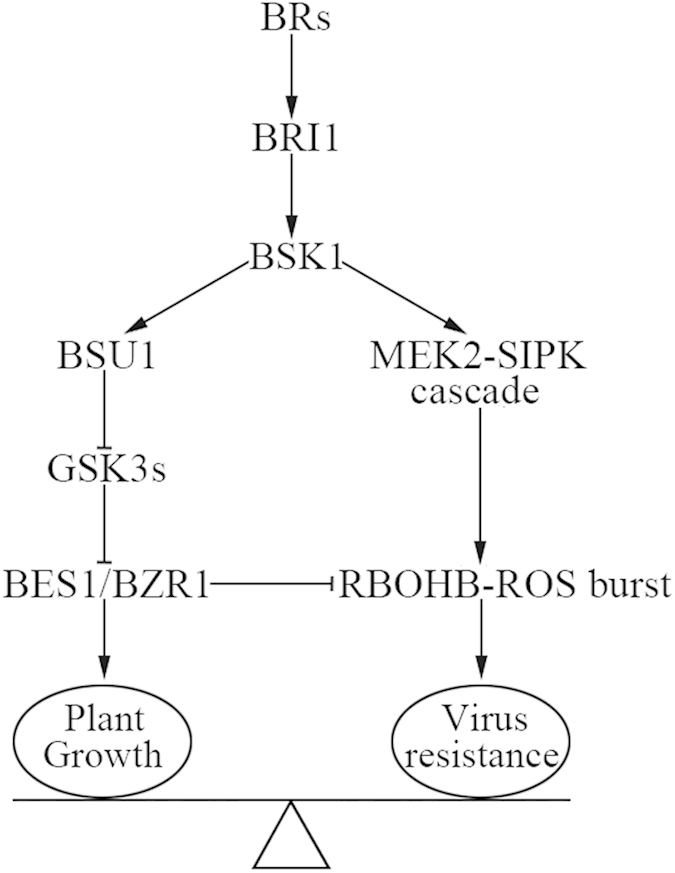
A proposed model for BR-mediated virus resistance. BSK1 is activated by BRs through BRI1, thus leads to activation of downstream signaling. On the one hand, activated BSK1 induces MEK2-SIPK cascade, which in turn activates RBOHB-dependent oxidative burst. Thus enhance plant resistance to virus. On the other hand, BRs activates BES1/BZR1 through BR signaling pathway, activated BES1/BZR1 inhibits RBOHB-dependent ROS production and promote plant growth. The growth-immunity trade-off depends on the relative accumulation of BES1/BZR1 induced by BRs.

A balance between growth and immunity exists in plants, and BRs have emerged as crucial regulators of the growth-immunity trade-off^55^. In addition to enhanced growth phenotypes, co-application of BL and Bikinin suppressed TMV-GFP resistance in *N. benthamiana* (Fig. S11). This result indicates that activation of BR signaling pathway downstream of GSK3-like kinases leads to inhibition of viral defense response. New evidence indicates that BRs suppression of immunity is mainly mediated by signal integration at the level of transcriptional regulation. The BR-activated transcription factor BZR1 is shown to directly regulate many defense related genes that negatively regulate immune responses^56^. In addition, the recently described bHLH transcription factor HBI1, which is activated in response to BR signaling, triggers repression of steady-state expression of genes encoding immune components^57^,^58^. In our study, silencing of *NbBES1/BZR1* impaired Bikinin-mediated suppression of BR-trigered *NbRBOHB* expression and ROS production (Figs 3 and 4), so the inhibition of RBOHB-dependent ROS burst by BES1/BZR1 might inhibit BR-mediated activation of virus resistance in *N. benthamiana*. Thus, we hypothesize that when BR activates BRI1, BSK1 is activated and dissociates from the BRI1 complex. Activated BSK1 seems to have two opposite effects on ROS-mediated defense response, and the outcome seems to depend on the relative levels of BES1/BZR1 activated by BRs. When active form of BES1/BZR1 is relatively low, RBOHB-dependent oxidative burst mediated by MEK2-SIPK cascade may exert a dominant effect of BRs on virus resistance. When activated BES1/BZR1 level is high, increased BR signaling would suppress RBOHB-dependent ROS production through BES1/BZR1 and promote plant growth (Fig. 9). Thus, BSK1 may be a branching point where BR-mediated growth signaling and defense signaling split. In the future, it would be of great interest to determine how BSK1 is mechanistically connected to the MAPK cascades and roles of GSK3-like kinases in virus resistance.

In summary, the present study confirms the roles of BRs in viral defense response and reveals potential mechanisms of BRs action in TMV resistance. We present evidence for the involvement of the trade-off between growth and immunity in BR signaling pathway in the modulation of virus resistance in *N. benthamiana*. Through loss-of-function and gain-of-function analyses, we demonstrate that the MEK2-SIPK cascade modulates the BR-induced RBOHB-dependent oxidative burst in response to virus infection. Thus, our study contributes to the understanding of signaling cascades mediated by BRs in response to virus, and provides insights into the molecular mechanisms of plant defense against virus pathogens.

## Materials and Methods

### Plant materials and growth conditions

The *N. benthamiana* plants were grown in a greenhouse at 25 °C and cycles of 16 h of light (100 μmol m^−2^ s^−1^) and 8 h of darkness. Seedlings used in the experiments were 5 to 6 weeks old.

### Chemical treatments and pathogen inoculation

Brassinolide (BL, the most active BR) and brassinazole (BRZ, a specific inhibitor of BR biosynthesis) were purchased from Wako Pure Chemical Industries, ltd (Chuo-Ku, Osaka, Japan) and Santa Cruz Biotechnology, inc (Dallas, Texas, USA), respectively. Bikinin was purchased from Sigma (St. Louis). The hormone and inhibitor solutions were prepared in water containing 0.02% (vol/vol) Tween 20. The chemicals and the concentrations used are as follows: BL (0.1 μM), BRZ (1 μM) and Bikinin (50 μM). Distilled water containing 0.02% (vol/vol) Tween 20 was used as a control treatment.

In infection experiments, the chemicals were sprayed 12 h before virus inoculation. Purified TMV-GFP RNA was maintained in an aqueous suspension of 0.02 M sodium phosphate buffer (PBS) at 4 °C. Three leaves of each *N. benthamiana* plant were inoculated with 0.1 μg of TMV-GFP RNA. PBS buffer without virus RNA was rubbed onto the leaves as the control experiment.

### TRV-mediated VIGS assay

VIGS was performed as described previously^2^. For construction of VIGS vectors, partial cDNA of *NbDWARF* (342 bp), *NbBRI1* (363 bp), *NbBAK1* (258 bp), *NbBSK1* (300 bp), *NbBIK1* (263 bp), *NbBSU1* (333 bp), *NbBES1/BZR1* (324 bp), *NbRBOHA* (278 bp), *NbRBOHB* (365 bp), *NbMEK1* (356 bp), *NbMEK2* (291 bp), *NbNTF6* (277 bp), *NbSIPK* (255 bp) and *NbWIPK* (273 bp) was amplified by RT-PCR from a cDNA library of *N. benthamiana* leaf tissues using gene specific primers (Table. S1), *NbRBOHA*&*RBOHB* (500 bp) were amplified through overlap-extension PCR. Then these PCR products were cloned into the TRV vector (pTRV2). For VIGS assay, pTRV1 or pTRV2 (with the inserted fragment) were introduced into *Agrobacterium* strain GV2260. A mixture of equal parts of *Agrobacterium* cultures containing of pTRV1 and pTRV2 or its derivatives was inoculated into the 4-leaf stage plants. To determine the efficiency of VIGS, RT-PCR was performed with primers targeting sites outside the cloned fragments in upper leaves at 12 dpi. VIGS experiments were repeated at least three times with more than six plants for each repeat.

### Agrobacterium-mediated transient expression

The full length cDNA fragment was amplified and inserted into the pBI121 vector, in which a *Flag*-tag was added to the C-terminal end. Then the recombinant plasmids were transformed into *Agrobacterium tumefaciens* strain EHA105 by the freeze-thaw method. *Agrobacterium tumefaciens* carrying each constructs were cultured overnight at 28 °C. Then, bacterial cells were harvested and resuspended in an infiltration buffer containing 10 mM MES (pH 5.6), 10 mM MgCl_2_, and 150 μM acetosyringone to a final OD_600_ of 1.0. After incubated for 3 h at room temperature, the bacterial suspensions were infiltrated onto the lower leaf surfaces of *N. benthamiana* plants with a syringe.

### GFP imaging

GFP fluorescence was photographed under UV light using a Canon G11 digital camera and a B-100AP long wave UV lamp (Ultra-Violet Products, USA).

### Superoxide, H_2_O_2_ staining and H_2_O_2_ determinations

Superoxide and H_2_O_2_ staining were visually detected with nitro blue tetrazolium (NBT) and 3,3'-diaminobenzidine (DAB). *N. benthamiana* leaves were vacuum infiltrated with NBT (0.5 mg/mL) solutions for 2 h or DAB (2 mg/mL) solutions for 8 h. Leaves were then decolorized in boiling ethanol (95%) for 15 min. H_2_O_2_ accumulation was determined using the Amplex red hydrogen peroxide/peroxidase assay kit (Invitrogen, USA).

### Determination of antioxidant enzymes

For the enzyme assays, 500 mg of leaves were homogenized in 5 ml 25 mM PBS buffer (PH = 7.8) containing 0.2 mM EDTA, 2 mM ascorbic acid and 2% PVP, with the addition of 1 mM ascorbate in the case of the Ascorbate peroxidase (APX) assay. The homogenate was centrifuged at 12,000 g for 20 min at 4 °C and the supernatant was immediately used for the determination of enzymatic activity. Superoxide dismutase (SOD) activity was assayed by measuring the ability to inhibit the photochemical reduction of NBT, one unit of SOD activity was defined as the amount of enzyme that was required to cause 50% inhibition of the reduction of nitro blue tetrazolium, as monitored at 560 nm. APX activity was measured by monitoring the decrease in absorbance at 290 nm as ascorbate was oxidized.

### RNA extraction and quantitative real-time PCR

Total RNA was extracted using Trizol Reagent (Invitrogen, USA) from *N. benthamiana* leaves according to the manufacturer’s recommendations. All RNA samples were treated with DNase I before PCR. For RT, the first-strand cDNA was prepared using the ReverTra Ace kit (Toyobo, Japan). To further assay the expression levels of genes, quantitative real-time PCR analysis was performed on a Bio-Rad iCycler (Bio-Rad, Beijing, China). Relative quantitation of the target gene expression level was performed using the comparative *C*_t_ (threshold cycle) method. At least three biological replicates were performed for each sample and three technical replicates were analyzed for each biological replicate. Amplification of *Actin* gene was used as an internal control. The primer sequences were shown in Table. S2.

### Protein extraction and western blotting analysis

Total proteins were extracted with extraction buffer (50 mM Tris-Cl [pH 6.8], 5% mercaptoethanol, 10% glycerol, 4% sodium dodecyl sulfate, and 4 M urea) in an ice bath. Protein concentrations were determined by the Bradford method using bovine serum albumin as a standard. For western blotting analysis, about 10 μg of protein from each sample were electrophoresed in 15% SDS-polyacrylamide gels and transferred to nitrocellulose membranes. Then the membranes were hybridized with anti-*TMV CP* or anti-*Flag* sera.

### Statistical analysis

Statistical analysis of the results from experiments with three or more mean values used a one-way analysis of variance (ANOVA) as dictated by the number of main effects. The difference was considered to be statistically significant when P < 0.05.

## Additional Information

**How to cite this article**: Deng, X.-G. *et al*. Role of brassinosteroid signaling in modulating *Tobacco mosaic virus* resistance in *Nicotiana benthamiana. Sci. Rep.* **6**, 20579; doi: 10.1038/srep20579 (2016).

## Supplementary Material

Supplementary Information
