# Supplementary material for: Role of brassinosteroid signaling in modulating Tobacco mosaic virus resistance in Nicotiana benthamiana
Source: Sci Rep. 2016 Feb 3;6:20579. doi: 10.1038/srep20579 (PMC4738339; doi:10.1038/srep20579)
Supplement: Supplementary Information [file srep20579-s1.doc]

**Supplementary Information**

**Role of brassinosteroid signaling in** **modulating *Tobacco mosaic virus* resistance in *Nicotiana benthamiana***

Xing-Guang Deng^1, a^, Tong Zhu^1^, Xing-Ji Peng^1^, De-Hui Xi^1^, Hongqing Guo^2^, Yanhai Yin^2^, Da-Wei Zhang^1, a, *^ & Hong-Hui Lin^1, *^

^1^Ministry of Education Key Laboratory for Bio-Resource and Eco-Environment, College of Life Science, State Key Laboratory of Hydraulics and Mountain River Engineering, Sichuan University, Chengdu, Sichuan, 610064, China

^2^Department of Genetics, Development and Cell Biology, Plant Science Institute, Iowa State University, Ames, IA 50011, USA.

^a^Authors who contributed equally to this work

^*^Corresponding author, E-mail: hhlin@scu.edu.cn and yuanmiao1892@163.com

Fax: +86 28 85415389

**Supplementary Table S1.** Primers used for construction of VIGS and over expression vectors.

| **Gene** | **F primer (5’→3’)** | **R primer (5’→3’)** |
| --- | --- | --- |
| *DWARF* | ACTGTTTCCACCACTTCT | GTGTTCCAGGCTCTTATC |
| *BRI1* | CTTGAAGATGTTCTGCACGA | CGTTCTGCCAGTTAGGAGC |
| *BAK1* | ACTTGATTGGGTGAAGGGAC | GGCTGGTGGACATGGTTGT |
| *BSU1* | TGGTGGGATAGGGAGGTCA | CCAGCATTGTTGGCGGTTC |
| *BSK1* | AAATGAGGCTGCTGGACTT | CTTAGAGTGGAGACGTGGG |
| *BIK1* | ATCTAATTGAATGGGCTAAG | ATCGGTACAGGTTCTACGA |
| *BES1/BZR1* | ACTTCAGCCAACATTACGC | AGCAGCAAAGAAAGGGATA |
| *RBOHA* | GCCGGTGGACATCCGATACG | TCGTCGGCGATCGAGATTCCG |
| *RBOHB* | AGAGCGTCGTACAGTGGTC | GTAGGAATTGGGCGTTTAT |
| *MEK1* | GCTGACGTAATCAGGCAACT | TCCTCCGACTGTATGTATGG |
| *MEK2* | CTTCTCCGAACTTGAGCGTAT | GCTGACTCTTTAGGGATGTGG |
| *NTF6* | CACTTCAGAGGCGGATTT | TCGAGGGAGCTGCTTTAC |
| *SIPK* | TTCTGCACAGGGACTTGA | CGTGATCTCTACCAGGAA |
| *WIPK* | CCGTTACGAAGGGAGTTTTC | TCCGTCATATTCTCGTTCTC |
| *SIPK-OE* | GG*GGTACC*ATGGATGGTTCTGGTCAGCAG (*KpnI*) | CG*GGATCC*TCACATGTGCTGGTATTCAG (*BamHI*) |

**Supplementary Table S2.** Primers used for quantitative real-time polymerase chain reaction (PCR) and RT-PCR analysis of gene expression.

| **Gene** | **F primer (5’→3’)** | **R primer (5’→3’)** |
| --- | --- | --- |
| *CPD* | ATTCCTCACCGAGACACCC | ATTGCTCTGCCACCTCCAC |
| *DWARF* | CTAGGCACTCTTTCACTAC | AGATATTTCACAGCCATCA |
| *BRI1* | GCTCCTAACTGGCAGAACG | GGAATCAATACCTGACCCT |
| *BRL1* | TGTGGTGGTATCAAATGCA | AGTTAAGTGAAGGCGAAGC |
| *BRL2* | GGTCCGTTTCCTGATTCTA | TGGTGCTCTTAGCTCTTCCA |
| *BSK1* | TTGTTGCTACACTTGCTCCAT | TGCCGTCTTGAACTCTTTA |
| *BSK2* | CCAAGAATGGACCCAACAA | GCATAGCATCCCTTAGAGC |
| *BSK3* | GGTCGTTGGGTTGCTGTTA | CCTCATCGCCCATTTCATA |
| *BAK1* | CCTCCTACACCCTCATCTT | CAACACTTCCGTTCTCCAT |
| *SERK2* | TACCGAGTGATCTTGGAAA | AAACTGATAGGCGTGAATA |
| *BIK1* | GGGTCTTGTATCAGTGTTCG | ATTTCTGCCAGCCATTCTT |
| *PBS1* | TCTTCAGGAGCAGACAGAC | GCATAAGCACCTCCACTAA |
| *BSU1* | CATCACCGAGGTATCAACA | CATAAGCTCCAAAGAGGGA |
| *BSL1* | AGGCGATAGACCGTCAGCTA | CGCATAAGCTCCAAAGAAGG |
| *BSL2* | TGCACGCAGTTATGGAGAAG | AACCAGCAGAATTCCCTTCA |
| *BES1/BZR1* | GGAAATAGTAGTAGTGGGAGG | ATAAGTAGTGCCATCAGGTT |
| *BEH2* | AGGATCGTCGGGAAGGTTG | TCCCTTGCGATAAGTGGTG |
| *BEH4* | GAGCGGGAGAATAATAAGA | TTGGGTTGTTGGAAGTGAA |
| *RBOHA* | ACAAATGTCTAAGCCTCCC | GCTCCATAAGGTCCATCTATT |
| *RBOHB* | AACAACCTCGGATACATTAT | TGTAAATAGACCAGCCATAA |
| *MEK1* | TATTGTTAGCAGTCCTCCAC | ACTCACAAGTATGCCGAAA |
| *MEK2* | GTGATTGGGCTAGTCTTAT | AGTGGCTGGTCTATTCTGG |
| *NTF6* | GCTATTAGGTTCGCCAGAG | ATCGCAAACAGGTTCCTC |
| *SIPK* | TATAATTCCACCACCACAGA | CTTCATCTGTTCCTCCGTAA |
| *WIPK* | CAATTCCCTGATTTTCCTTCGG | GGAAAGTAGATACTCCAGATC |
| *PR1* | TGCCTTCATTTCTTCTTG | TTAGTATGGACTTTCGCCTCT |
| *PR2* | CTAATGGCATCAGAAAGA | ATTGGCTAAGAGTGGAAG |
| *HMGR2* | CATTGGAGGGATTTGATTAT | ACAAGGTGCTCTGGTCATT |
| *EDS1* | ATTATCAGCACGAGGTAGTTG | ACCAGTTTCCCATTTCCAG |
| *CAT1* | AAGCCAAATCCTAAGTCCC | CTGCCTCATCTTCCAACAA |
| *GST* | ATCCCTCGTTATTGTTCAG | CAAAGTATGGCTTGTCTCC |
| *ICS1* | TTAAACTCATCATCTTCAG | GGCTTCGCCGGCATTCATT |
| *ACTIN* | AACTGATGAAGATACTCACA | CAGGATACGGGGAGCTAAT |
| TMV*-CP* | TCTTGTCATCAGCGTGGGC | GCTGTGACTAGCGGGTCTA |

**
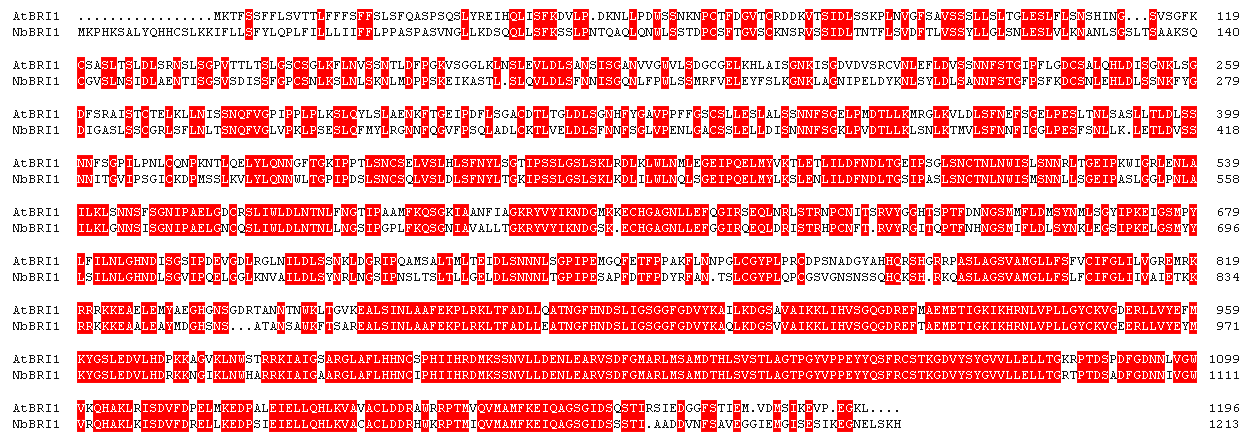
**

**Supplementary Figure S1.** Alignment of BRI1 protein sequences from *Arabidopsis* and *N. benthamiana*. Sequences were aligned using DNAMAN, pairwise comparisons showed 64.13% identity between AtBRI1 and NbBRI1 at amino acid level.


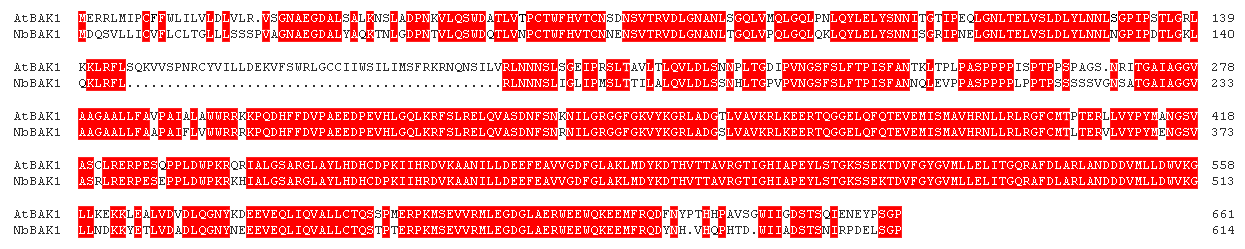


**Supplementary Figure S2.** Alignment of BAK1 protein sequences from *Arabidopsis* and *N. benthamiana*. Sequences were aligned using DNAMAN, pairwise comparisons showed 77.26% identity between AtBAK1 and NbBAK1 at amino acid level.


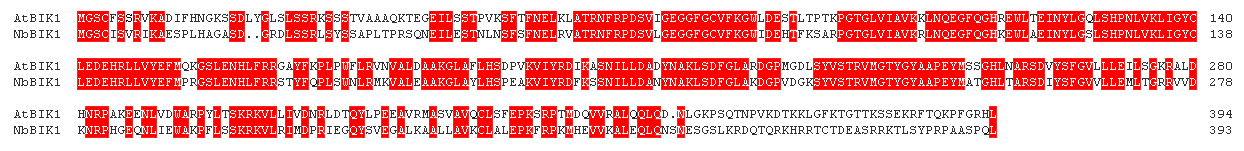


**Supplementary Figure S3.** Alignment of BIK1 protein sequences from *Arabidopsis* and *N. benthamiana*. Sequences were aligned using DNAMAN, pairwise comparisons showed 62.12% identity between AtBIK1 and NbBIK1 at amino acid level.


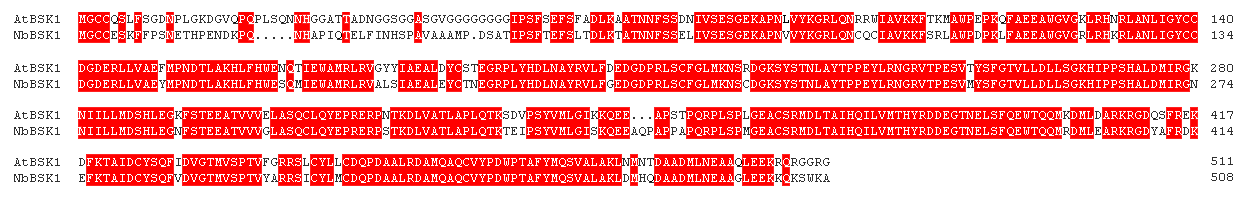


**Supplementary Figure S4.** Alignment of BSK1 protein sequences from *Arabidopsis* and *N. benthamiana*. Sequences were aligned using DNAMAN, pairwise comparisons showed 79.42% identity between AtBSK1 and NbBSK1 at amino acid level.

**
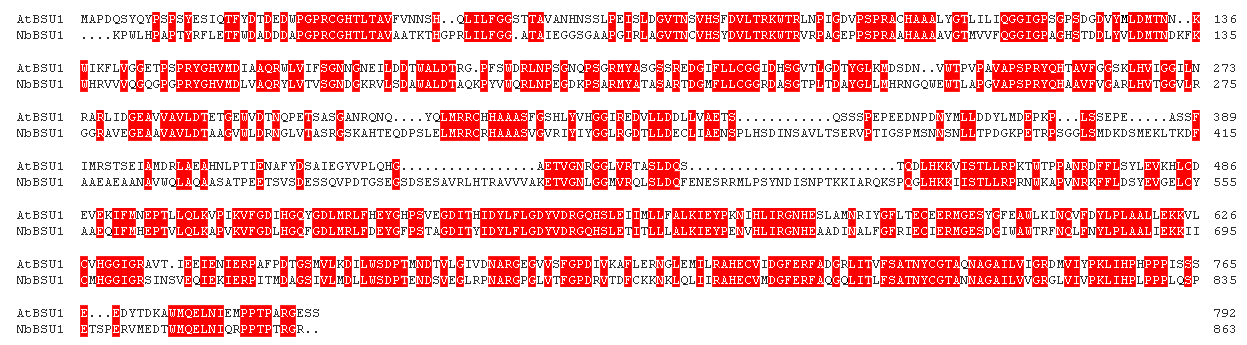
**

**Supplementary Figure S5.** Alignment of BSU1 protein sequences from *Arabidopsis* and *N. benthamiana*. Sequences were aligned using DNAMAN, pairwise comparisons showed 52.58% identity between AtBSU1 and NbBSU1 at amino acid level.


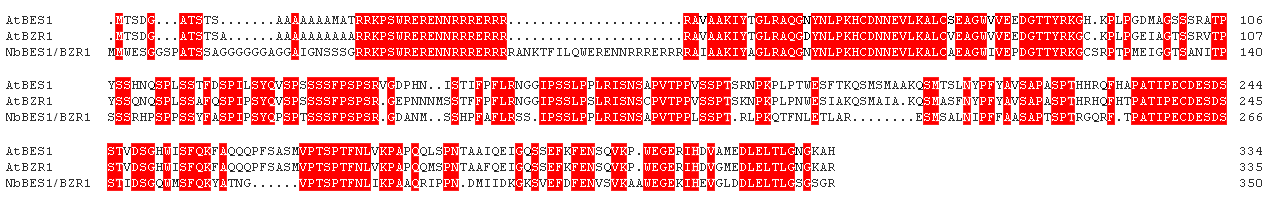


**Supplementary Fig S6.** Alignment of BES1/BZR1 protein sequences from *Arabidopsis* and *N. benthamiana*. Sequences were aligned using DNAMAN. Pairwise comparison showed 75.09% identity between AtBES1/BZR1 and NbBES1/BZR at amino acid level.


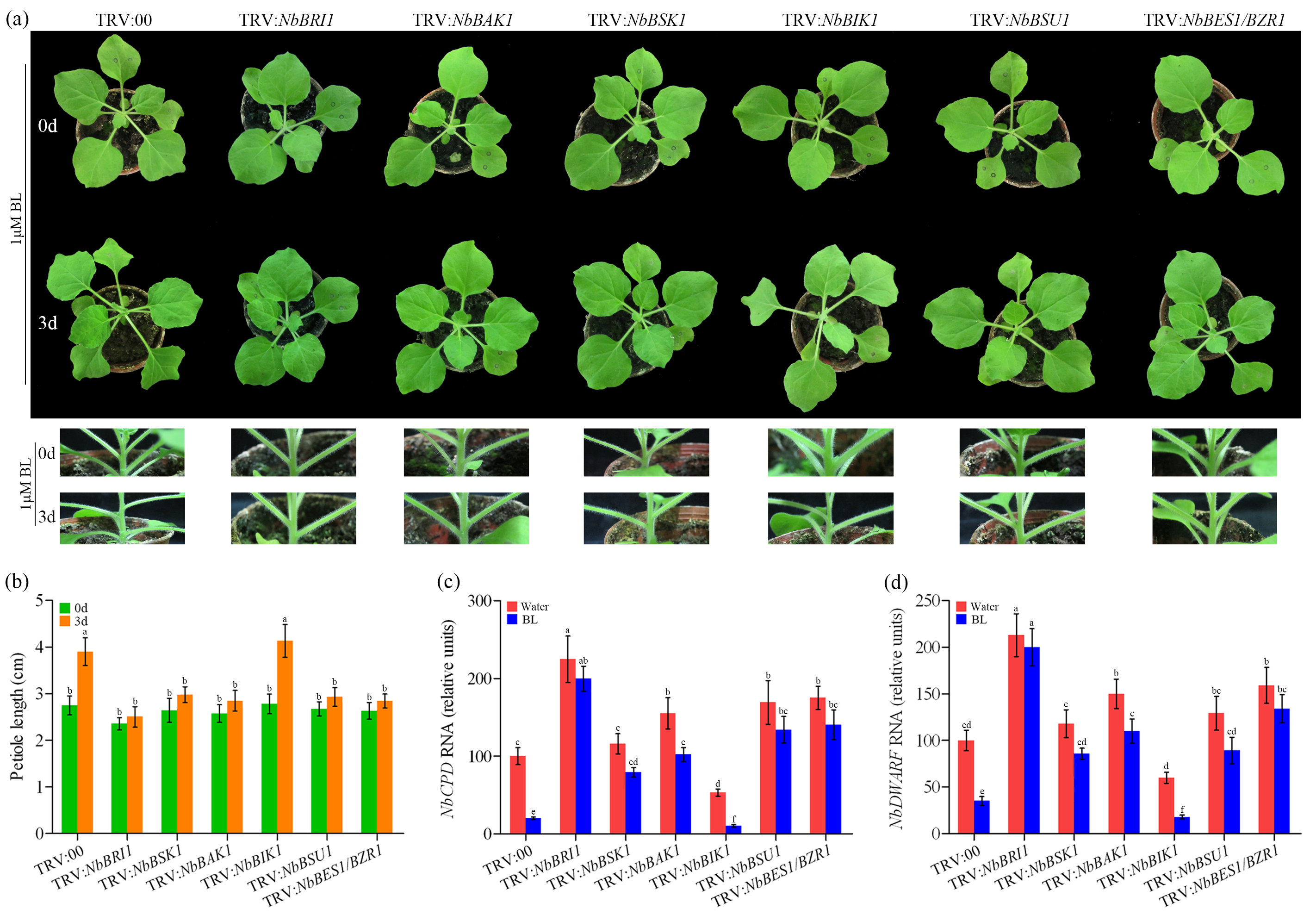


**Supplementary Figure S7.** *NbBRI1*, *NbBSK1*, *NbBAK1*, *NbBIK1*, *NbBSU1* and *NbBES1/BZR1* are involved in the BRs response in *N. benthamiana*. (a) Growth phenotypes and leaf angles comparison of control and gene silenced plants. *N. benthamiana* plants were treated with 1 μM BL and pictures were taken at 0 day or 3 days treatments. (b) Petiole lengths of the seventh leaves in control or gene silenced *N. benthamiana* plants. Quantitative real-time PCR analysis of *NbCPD* (c) and *NbDWARF* (d) expression in control or gene silenced plants after one day of BL treatment. Bars represent mean and standard deviation of values obtained from three biological replicates per genotype and time point. Significant differences (P<0.05) are denoted by different lowercase letters.

**
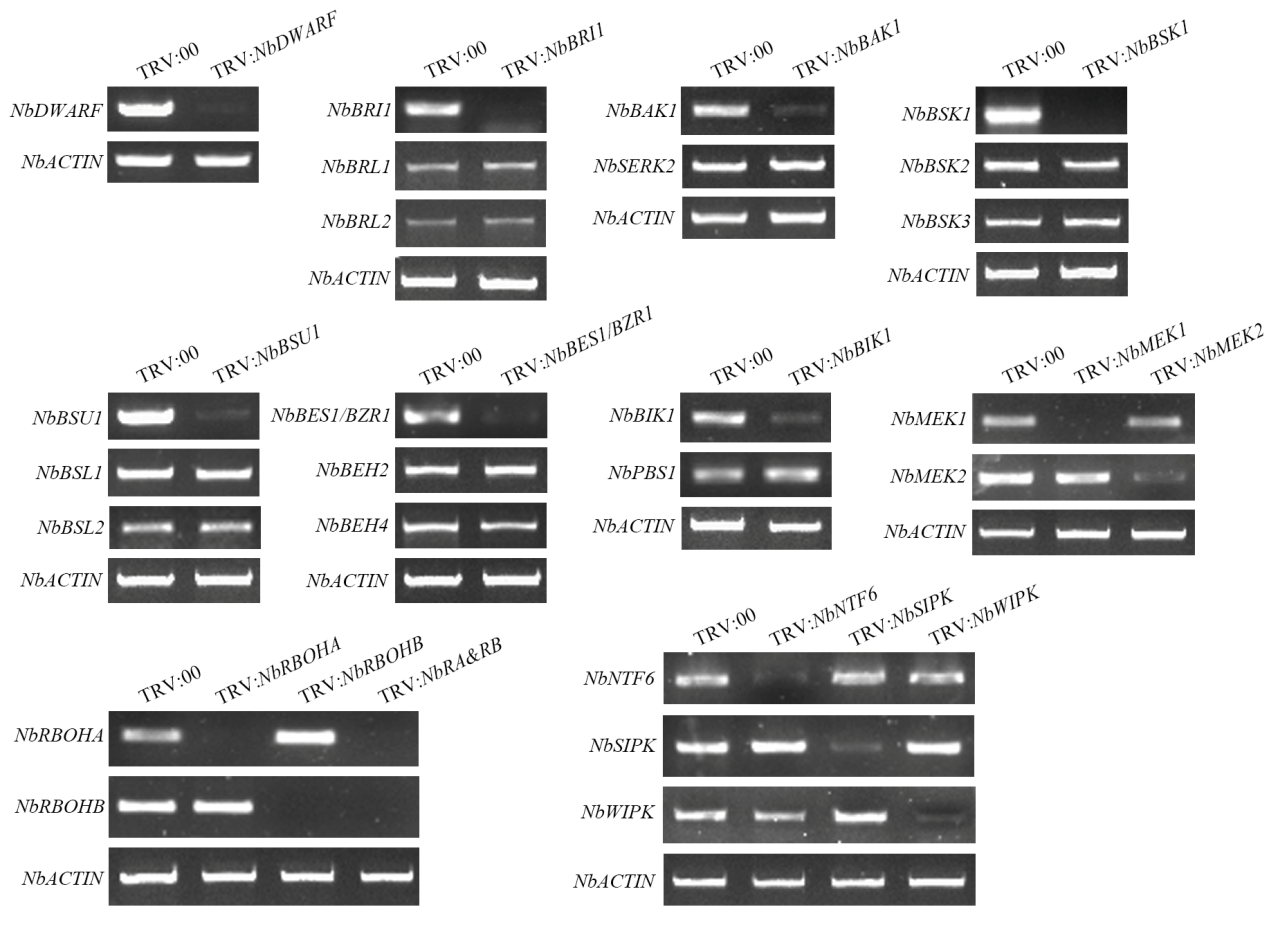
**

**Supplementary Figure S8.** Confirmation of the efficiency and specificity of gene silencing in *N. benthamiana* plants. RT-PCR analysis was conducted using total RNA extracted from leaves, of plants inoculated with *Agrobacterium* GV2260 carrying TRV-target genes and the corresponding non-silenced leaves of TRV:00 infected control plants. Typical PCR products are also shown for *NbACTIN*, used as an internal standard to correct the quantity, from the same tissues.


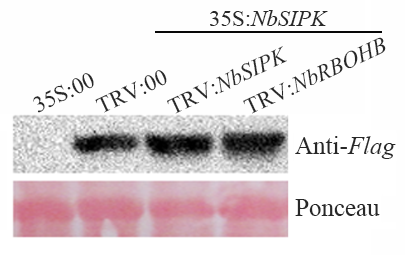


**Supplementary Figure S9.** Western blotting analysis of *NbSIPK* expression in *N. benthamiana* leaves described in Fig 7. Rubisco proteins were used as loading controls and stained with Ponceau.


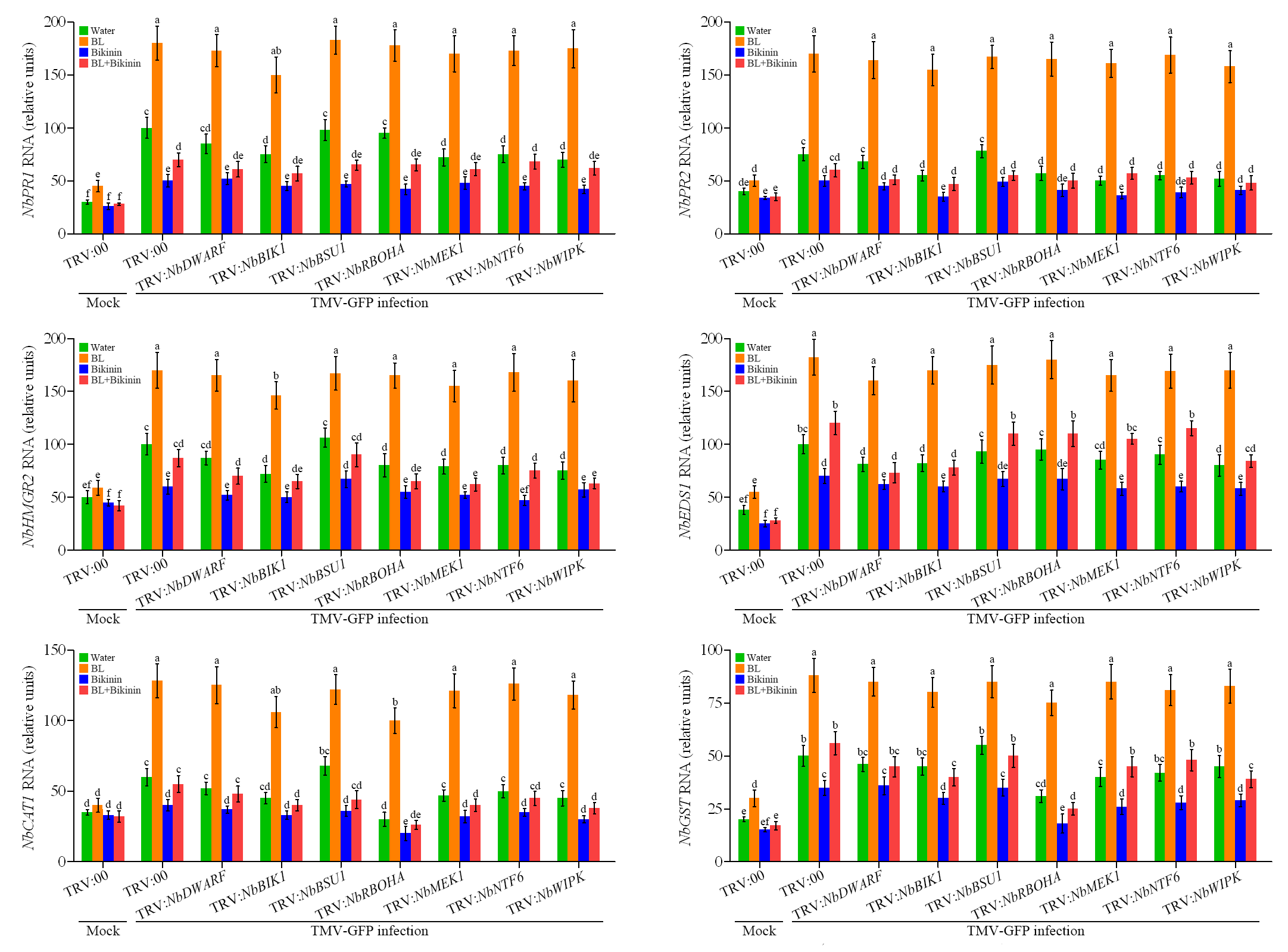


**Supplementary Figure S10.** Relative expression of disease-related genes (*PR1*, *PR2*, *HMGR2* and *EDS1*) and antioxidant-related genes (*CAT1* and *GST*) in *NbDWARF*-, *NbBIK1*-, *NbBSU1*-, *NbRBOHA*-, *NbMEK1*-, *NbNTF6*-, *NbWIPK*-silenced and control (TRV:00) *N. benthamiana* plants at 2 days post inoculation (dpi) with TMV-GFP infection, these plants were pretreated with water, BL, Bikinin or BL+Bikinin. “Mock” means seedlings not infected with TMV-GFP. Bars represent mean and standard deviation of values obtained from three biological replicates per genotype and time point. Significant differences (P<0.05) are denoted by different lowercase letters.


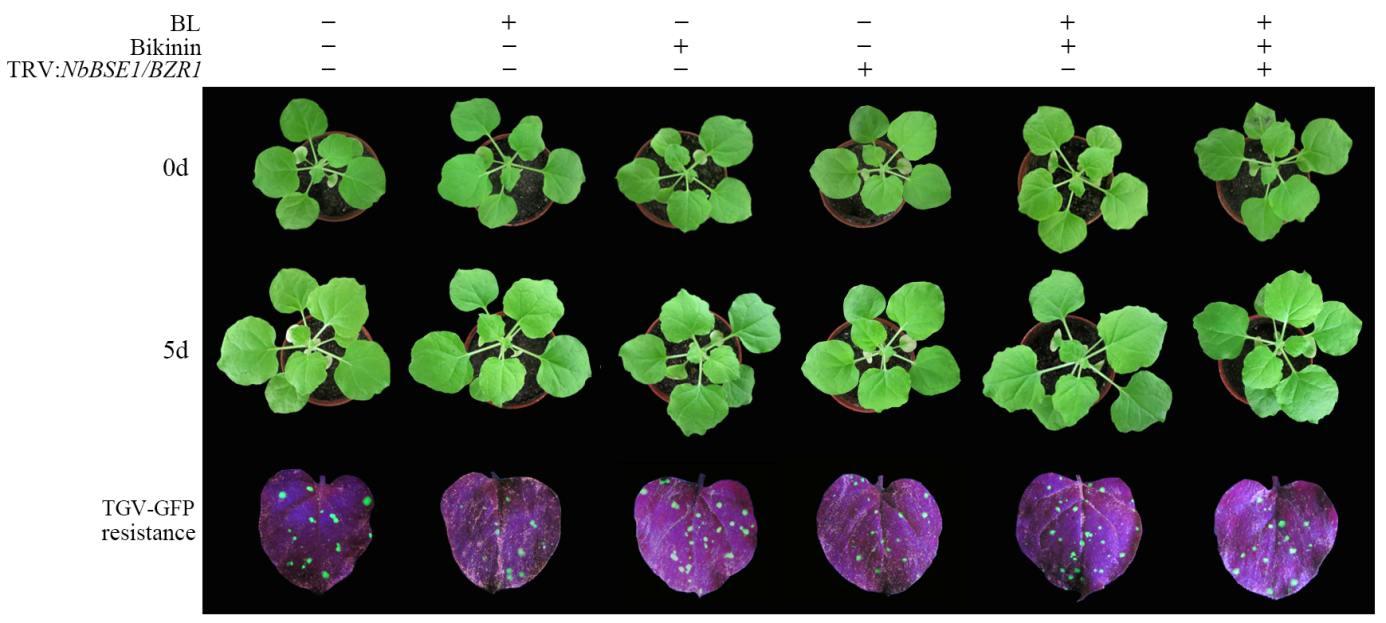


**Supplementary Figure S11.** Effects of 0.1 μM BL, 50 μM Bikinin, silencing of *NbBES1/BZR1* or combined treatment on plant growth and TMV-GFP resistance in *N. benthamiana*. Pictures of *N. benthamiana* phenotypes were taken at 0 day or 5 days treatments. Pictures of *N. benthamiana* inoculated leaves were taken under a UV light at 5 days post inoculation (dpi).
